# Circulating insulin‐like growth factor‐I, total and free testosterone concentrations and prostate cancer risk in 200 000 men in UK Biobank

**DOI:** 10.1002/ijc.33416

**Published:** 2020-12-11

**Authors:** Eleanor L. Watts, Georgina K. Fensom, Karl Smith Byrne, Aurora Perez‐Cornago, Naomi E. Allen, Anika Knuppel, Marc J. Gunter, Michael V. Holmes, Richard M. Martin, Neil Murphy, Konstantinos K. Tsilidis, Bu B. Yeap, Timothy J. Key, Ruth C. Travis

**Affiliations:** ^1^ Cancer Epidemiology Unit, Nuffield Department of Population Health University of Oxford Oxford UK; ^2^ Genetic Epidemiology Group International Agency for Research on Cancer Lyon France; ^3^ Clinical Trial Service Unit and Epidemiological Studies Unit, Nuffield Department of Population Health University of Oxford Oxford UK; ^4^ UK Biobank Ltd Stockport UK; ^5^ Section of Nutrition and Metabolism International Agency for Research on Cancer Lyon France; ^6^ Medical Research Council Population Health Research Unit University of Oxford Oxford UK; ^7^ MRC Integrative Epidemiology Unit (IEU), Population Health Sciences, Bristol Medical School University of Bristol Bristol UK; ^8^ Bristol Medical School, Department of Population Health Sciences University of Bristol Bristol UK; ^9^ National Institute for Health Research (NIHR) Bristol Biomedical Research Centre University Hospitals Bristol NHS Foundation Trust and the University of Bristol Bristol UK; ^10^ Department of Hygiene and Epidemiology University of Ioannina School of Medicine Ioannina Greece; ^11^ Department of Epidemiology and Biostatistics, School of Public Health Imperial College London London UK; ^12^ Medical School University of Western Australia Perth Australia; ^13^ Department of Endocrinology and Diabetes Fiona Stanley Hospital Perth Australia

**Keywords:** IGF‐I, Mendelian randomisation, prospective analysis, prostate cancer, testosterone

## Abstract

Insulin‐like growth factor‐I (IGF‐I) and testosterone have been implicated in prostate cancer aetiology. Using data from a large prospective full‐cohort with standardised assays and repeat blood measurements, and genetic data from an international consortium, we investigated the associations of circulating IGF‐I, sex hormone‐binding globulin (SHBG), and total and calculated free testosterone concentrations with prostate cancer incidence and mortality. For prospective analyses, risk was estimated using multivariable‐adjusted Cox regression in 199 698 male UK Biobank participants. Hazard ratios (HRs) were corrected for regression dilution bias using repeat hormone measurements from a subsample. Two‐sample Mendelian randomisation (MR) analysis of IGF‐I and risk used genetic instruments identified from UK Biobank men and genetic outcome data from the PRACTICAL consortium (79 148 cases and 61 106 controls). We used *cis*‐ and all (*cis* and *trans*) SNP MR approaches. A total of 5402 men were diagnosed with and 295 died from prostate cancer (mean follow‐up 6.9 years). Higher circulating IGF‐I was associated with elevated prostate cancer diagnosis (HR per 5 nmol/L increment = 1.09, 95% CI 1.05‐1.12) and mortality (HR per 5 nmol/L increment = 1.15, 1.02‐1.29). MR analyses also supported the role of IGF‐I in prostate cancer diagnosis (*cis*‐MR odds ratio per 5 nmol/L increment = 1.34, 1.07‐1.68). In observational analyses, higher free testosterone was associated with a higher risk of prostate cancer (HR per 50 pmol/L increment = 1.10, 1.05‐1.15). Higher SHBG was associated with a lower risk (HR per 10 nmol/L increment = 0.95, 0.94‐0.97), neither was associated with prostate cancer mortality. Total testosterone was not associated with prostate cancer. These findings implicate IGF‐I and free testosterone in prostate cancer development and/or progression.

## Abstract 2

**What's new?**

Testosterone, insulin‐like growth factor‐I (IGF‐I), and sex hormone‐binding globulin (SHBG) all have been associated with prostate‐cancer risk. In this large, prospective study, the authors analyzed how these circulating hormones might impact mortality as well as risk. They found that men with higher IGF‐I had a higher risk of both prostate‐cancer diagnosis and mortality. Men with higher free testosterone had an increased risk of prostate cancer, while men with higher SHBG had a decreased risk. These results support the roles of IGF‐I and testosterone in prostate cancer development.

**Abbreviations**
CI: confidence interval
GWAS: genome‐wide association study
HR: hazard ratio
ICD‐10: International Classification of Diseases Tenth Revision Code 10
IGF‐I: insulin like growth factor‐I
LD: linkage disequilibrium
MR: Mendelian randomisation
OR: odds ratio
PSA: prostate‐specific antigen
SHBG: sex hormone‐binding globulin
SNP: single nucleotide polymorphism

## 1 INTRODUCTION

Prostate cancer is the second most common cancer in men worldwide and a leading cause of cancer death.^1^ Few potentially modifiable risk factors have been identified, but circulating hormone concentrations are thought to play a role in prostate cancer aetiology.^2^, ^3^

Insulin‐like growth factor‐I (IGF‐I) is involved in cell proliferation, differentiation and apoptosis, and prospective studies have shown a positive association of circulating IGF‐I concentration with prostate cancer risk.^4^ Less is known about its potential role in prostate cancer progression or mortality.^5^

Androgens are integral to the maintenance of normal prostate function.^6^ In the circulation, testosterone is bound to sex hormone‐binding globulin (SHBG) and albumin. Approximately 2% of total testosterone circulates unbound or “free” and is postulated to be biologically active.^7^ Observational pooled analysis of individual participant data from prospective studies indicated that men with very low free testosterone may have a lower risk of prostate cancer,^8^ and a recent Mendelian randomisation (MR) study supports a positive association between free testosterone concentration and prostate cancer diagnosis.^9^ However, it is unclear whether circulating free testosterone concentration is associated with prostate cancer mortality.^8^, ^10^ Epidemiological studies have also reported an inverse association between prostate cancer risk and circulating SHBG,^8^ although results from MR analyses are inconclusive.^9^

Previous risk estimates for prostate cancer in relation to hormone concentration have generally been based on data from nested case‐control studies with a single blood draw at baseline. The UK Biobank study has standardised measurements of hormones from baseline blood samples collected in the whole cohort (500 000 participants) as well as repeat measurements of the hormones in a subset (20 000).

In this article, we aimed to examine the associations of serum concentrations of IGF‐I, SHBG, total and free testosterone with prostate cancer incidence and mortality, using observational data from UK Biobank. For IGF‐I, we investigated potential causal associations of IGF‐I with prostate cancer using MR analyses, with genetic data from UK Biobank and the PRACTICAL consortium (based on 79 000 prostate cancer cases and 61 000 controls). MR analyses of SHBG, total and free testosterone and prostate cancer risk using these datasets have recently been published.^9^ MR uses germline genetic variants as proxies of putative risk factors and estimates their associations with disease. As germline genetic variants are fixed and randomly allocated at conception, this technique minimises the possibility of confounding and reverse causality, and is therefore considered a useful approach towards causal inference.^11^ By using these two complementary approaches, we were able to robustly investigate associations and assess causation.

## 2 PATIENTS AND METHODS

### 2.1 UK Biobank observational analysis

#### 2.1.1 Study design

UK Biobank is a prospective cohort with open access for public health research. Details of the study protocol and data collection are available online (http://www.ukbiobank.ac.uk/wp-content/uploads/2011/11/UK-Biobank-Protocol.pdf) and elsewhere.^12^, ^13^

In brief, all participants were registered with the UK National Health Service (NHS) and lived within 40 km of one of the UK Biobank assessment centres. Approximately 9.2 million people were initially invited to participate. Overall, 503 317 men and women aged 40 to 69 years consented to join the cohort and attended one of 22 assessment centres throughout England, Wales and Scotland between 2006 and 2010, a participation rate of 5.5%.^13^

#### 2.1.2 Baseline assessment

At the baseline assessment visit, participants provided information on a range of sociodemographic, physical, lifestyle and health‐related factors via a self‐completed touchscreen questionnaire and a computer‐assisted personal interview.^13^ Weight and height were measured at the assessment centre.^13^

#### 2.1.3 Blood sampling and biomarker assays

At recruitment, blood sampling was successfully performed in 99.7% of the cohort. Blood was collected in a serum separator tube and shipped to the central processing laboratory in temperature‐controlled boxes at 4°C,^14^ then aliquoted and stored in a central working archive at −80°C.^15^ Serum concentrations of circulating IGF‐I, SHBG, testosterone and albumin were measured in all participants. IGF‐I (DiaSorin Liaison XL), SHBG and testosterone (Beckman Coulter AU5800) were determined by chemiluminescent immunoassays. Albumin was measured by a colorimetric assay (Beckman Coulter AU5800). Average within‐laboratory (total) coefficients of variation for low, medium and high internal quality control level samples for each biomarker ranged from 2.1% to 8.3%. Full details of the assay methods and quality assurance protocols are available online (https://biobank.ndph.ox.ac.uk/showcase/docs/serum_biochemistry.pdf).

#### 2.1.4 Free testosterone estimation

Free testosterone concentrations were estimated using a formula based on the law of mass action from measured total testosterone, SHBG and albumin concentrations.^16^, ^17^

#### 2.1.5 Repeat assessment

Participants who lived within a 35 km radius were invited to attend a repeat assessment clinic at the UK Biobank Co‐ordinating Centre in Stockport between August 2012 and June 2013. Repeat assessments were completed in 20 000 participants (9000 men) with a response rate of 21%.^18^

#### 2.1.6 Participant follow‐up

Cancer registration data were provided via record linkage to the NHS Central Register and obtained via NHS Digital, until the censoring date (31 March 2016 in England and Wales and 31 October 2015 in Scotland). Death data for England and Wales were provided by NHS Digital and for Scotland by the Information and Statistics Division (censoring dates 31 January 2018 in England and Wales, and 30 November 2016 in Scotland). In the analysis of incident prostate cancer, the endpoint was defined as the first diagnosis of prostate cancer, or prostate cancer mortality (primary or otherwise) (International Classification of Diseases Tenth revision code [ICD‐10] C61^19^), whichever was recorded first. In the analysis of prostate cancer mortality, the endpoint was prostate cancer as the primary cause of death. Person‐years were calculated from the date of recruitment to the date of the first cancer registration (excluding non‐melanoma skin cancer [ICD‐10 C44]), death or censoring date, whichever occurred first.

#### 2.1.7 Exclusion criteria

Our analytical dataset included 199 698 men; we excluded 9871 men with prevalent cancer (except C44: non‐melanoma skin cancer), 13 509 men who did not have blood data available or who had biomarker measurements that did not pass quality control procedures,^20^ 1685 participants for whom it was not possible to determine genetic sex or who were identified as being genetically female, 2326 men who reported taking hormone medication at baseline and 758 men who had no body mass index (BMI) data.

#### 2.1.8 Statistical analysis

Hazard ratios (HRs) and 95% confidence intervals (CIs) of prostate cancer diagnosis and mortality were estimated using Cox proportional hazards models, with age as the underlying time variable. Analyses were stratified by geographic area (10 UK regions) and age at recruitment (<45, 45‐49, 50‐54, 55‐59, 60‐64, ≥65 years), and adjusted for Townsend deprivation score (fifths, unknown [0.1%]), racial/ethnic group (white, mixed background, Asian, black, other and unknown [0.5%]), height (<170, ≥170‐<175, ≥175‐<180, ≥180 cm and unknown [0.1%]), lives with a wife or partner (no, yes), body mass index (BMI) (<25, ≥25‐<30, ≥30‐<35, ≥35 kg/m^2^), cigarette smoking (never, former, current light smoker (1‐<15 cigarettes per day), current heavy smoker (≥15 cigarettes per day), current (number of cigarettes per day unknown) and smoking status unknown [0.6%]), alcohol consumption (non‐drinkers, <1‐<10, ≥10‐<20, ≥20 g ethanol/day, unknown [0.5%]), and self‐reported diabetes (no, yes and unknown [0.5%]). Adjustment covariates were defined a priori based on previous analyses of UK Biobank data and categories were used to allow for nonlinear associations.^21^

Blood biomarker measurements were also available for up to 7776 men who attended a repeat assessment clinic a median of 4.4 years after first blood collection.^18^ Measurement error and within person variability using single measures at baseline leads to underestimation of risk (ie, regression dilution bias),^22^ to provide more precise and generalisable risk estimates, HRs for trend were estimated per absolute increase in usual hormone concentrations, with correction for regression dilution bias using the McMahon‐Peto method.^22^, ^23^

In the categorical analyses, biomarker measurements were categorised into fifths based on the distribution in the whole cohort and HRs were calculated relative to the lowest fifth of each blood parameter. The variance of the log risk in each group was calculated (from the variances and covariances of the log risk) and used to obtain group‐specific 95% CIs, which enable comparisons across different exposure categories.^24^

The proportional hazards assumption was examined using time‐varying covariates and Schoenfeld residuals and revealed no evidence of deviation.

#### 2.1.9 Subgroup analyses

Subgroup analyses for incident prostate cancer were examined by the following categories: age at diagnosis (≤65, >65 years), time from blood collection to diagnosis (≤4, >4 years), age at blood collection (<60, ≥60 years), BMI (<30, ≥30 kg/m^2^), smoking status (never or former, current), alcohol consumption (<10, ≥10 g ethanol/day), education status (no university degree, university degree), currently married/cohabiting (no, yes), Townsend index (<median, ≥median), ethnicity (white, non‐white), height (≤175, >175 cm), diabetes (no, yes), family history of prostate cancer (no, yes), poor self‐rated health (no, yes) and median observed hormone concentrations (<median, ≥median). Subgroup categories were chosen a priori on the basis of data distributions and previous analyses by this research group.^4^, ^8^ Heterogeneity in the associations for case‐specific variables (ie, age at diagnosis and time from blood collection to diagnosis) was examined using stratified Cox models based on competing risks and comparing the risk coefficients and standard errors in the two subgroups, and testing with a *χ* ^2^ for heterogeneity. For non‐case‐specific factors, heterogeneity was assessed using a *χ* ^2^ interaction term. Heterogeneity in the associations with prostate cancer mortality was not tested due to the limited statistical power.

#### 2.1.10 Further analyses

We examined the association of IGF‐I with incident prostate cancer after additional adjustment for concentrations of free testosterone and SHBG, and the associations of total and free testosterone and SHBG after further adjustment for IGF‐I (with adjustment for biomarkers categorised into fifths and unknown). As a further examination of trend, the categorical variable representing the fifths of the hormones was replaced with a continuous variable that was scored as 0, 0.25, 0.5, 0.75 and 1, such that a unit increase in this variable can be taken to represent an 80 percentile increase in concentrations to enable comparison across hormones and with previous pooled analyses.^4^, ^8^ Analyses with prostate cancer diagnosis were repeated after hormone concentrations were divided into tenths.

### 2.2 Mendelian randomisation analyses

#### 2.2.1 Genetic associations with circulating IGF‐I

Single nucleotide polymorphisms (SNPs) associated with circulating IGF‐I (*P* < 5 × 10^−8^ significance threshold) were identified from a publicly available genome wide association study (GWAS) from 167 174 male UK Biobank participants of European ancestry.^25^ SNPs were pruned by a linkage disequilibrium (LD) threshold of *r* ^2^ < 0.001. UK Biobank genotyping details are reported elsewhere.^26^

GWAS results were partitioned into one primary *cis‐*SNP instrument within the IGF‐I gene region on chromosome 12 (rs5742653), and 121 additional *trans‐*SNPs (SNPs associated with circulating IGF‐I concentrations that are not located in this gene region). These *cis* and *trans‐*SNPs together explained 6.3% of the variance in circulating concentrations of IGF‐I.^27^ SNP rs numbers, nearest gene and effect estimates are displayed in Supplementary Table S1.

#### 2.2.2 Genetic instruments for prostate cancer

We used summary statistics for SNP associations with prostate cancer risk that were generated from 79 148 prostate cancer cases and 61 106 controls of European ancestry from the PRACTICAL, CRUK, CAPS, BPC3 and PEGASUS consortia.^28^, ^29^ In brief, 44 825 prostate cancer cases and 27 904 controls were genotyped using OncoArray (http://epi.grants.cancer.gov/oncoarray/), and data were also available from several previous prostate cancer GWAS: UK stage 1 and stage 2; CaPS 1 and CaPS 2; BPC3; NCI PEGASUS; and iCOGS. Genotype information was imputed for all samples using the October 2014 release of the 1000 Genomes Project data as the reference panel. Odds ratios (ORs) and SEs were estimated using logistic regression and then meta‐analysed using an inverse variance fixed‐effect approach.

Where genetic instruments in the two datasets were not identical, we used HaploReg^30^ to identify SNPs in linkage disequilibrium (*r* ^2^ > 0.8) to use as proxies.

#### 2.2.3 Statistical analysis

We used a two‐sample MR approach to estimate IGF‐I associations with overall prostate cancer risk, using UK Biobank as our genetic instruments for IGF‐I and PRACTICAL for genetic outcome analyses.

The MR estimation for IGF‐I and prostate cancer was conducted by the Wald ratio using the *cis*‐SNP (rs5742653). We also conducted analyses incorporating all 122 IGF‐I associated SNPs using the inverse‐variance weighted method, as well as weighted median and mode‐based methods to reduce the influence of pleiotropy.^31^ The MR pleiotropy residual sum and outlier (MR‐PRESSO) test was used to investigate the role of outliers.^32^ To further assess the potential presence of horizontal pleiotropy, we used Cochran's *Q* for heterogeneity^31^ and the intercept from the MR‐Egger method. Additionally, we used leave‐one‐out analyses to test the sensitivity of our results to single SNP effects. PhenoScanner was used to assess pleiotropy of the genetic instruments.^33^, ^34^

All analyses were performed using Stata version 14.1 (Stata Corporation, College Station, TX) and R version 3.2.3. All tests of significance were two‐sided, and *P* values <.05 were considered statistically significant. MR analyses were performed using the *TwoSampleMR* R package.^35^

## 3 RESULTS

### 3.1 UK Biobank observational analyses

After a mean follow‐up of 6.9 years (SD = 1.3 years), 5402 (2.7%) men were diagnosed with prostate cancer and 295 died from the disease. Table 1 summarises the baseline characteristics of study participants. Mean age at recruitment was 56.5 years (SD = 8.2), and mean BMI was 27.8 kg/m^2^. 29% reported having had a prostate‐specific antigen (PSA) test prior to baseline and 14% had a family history of prostate cancer. Means and SDs for baseline biomarker measurements are displayed in Table 1. Regression dilution ratios ranged between 0.57 (free testosterone) and 0.80 (IGF‐I) (Supplementary Table S2).

**TABLE 1:** Baseline characteristics and blood data for all men and for men who developed prostate cancer in UK Biobank

	All men (*N* = 199 698)	Men who developed prostate cancer (*N* = 5402)
**Sociodemographic**		
Age at recruitment (years), mean (SD)	56.5 (8.19)	62.1 (5.26)
Most deprived quintile, % (*N*)	19.7 (39280)	15.8 (854)
Black ethnicity, % (*N*)	1.45 (2882)	2.18 (117)
Not in paid/self‐employment, % (*N*)	38.3 (76448)	56.7 (3062)
Living with partner, % (*N*)	92.9 (152616)	95.5 (4281)
**Anthropometric, mean (SD)**		
Height (cm)	175.7 (6.84)	175.1 (6.68)
BMI (kg/m^2^)	27.8 (4.23)	27.5 (3.79)
**Lifestyle, % (*N*)**		
Current cigarette smokers	12.5 (24855)	9.35 (502)
Drinking alcohol ≥20 g per day	43.7 (86905)	42.7 (2298)
Low physical activity (0–10 METs per week)	28.1 (54408)	26.6 (1393)
**Health history, % (*N*)**		
Hypertension	52.2 (104099)	58.6 (3163)
Diabetes	6.82 (13545)	5.91 (318)
Poor self‐rated health	4.81 (9547)	3.14 (169)
**Prostate‐specific factors, % (*N*)**		
Ever had a PSA test	28.7 (54139)	46.5 (2396)
Family history of prostate cancer	13.6 (14925)	25.0 (703)
**Baseline blood measures, mean (SD)**		
IGF‐I (nmol/L)	21.9 (5.52)	21.6 (5.23)
SHBG (nmol/L)	39.5 (16.6)	41.9 (16.0)
Total testosterone (nmol/L)	12.0 (3.65)	12.0 (3.53)
Free testosterone (pmol/L)	209 (59.5)	200 (54.5)
Albumin (g/L)	45.6 (2.61)	45.2 (2.53)

Abbreviations: BMI, body mass index; IGF‐I, insulin‐like growth factor‐I; METs, metabolic equivalent of tasks; PSA, prostate‐specific antigen; SHBG, sex hormone‐binding globulin.

#### 3.1.1 Associations between hormone concentrations and prostate cancer risk

**FIGURE 1:**
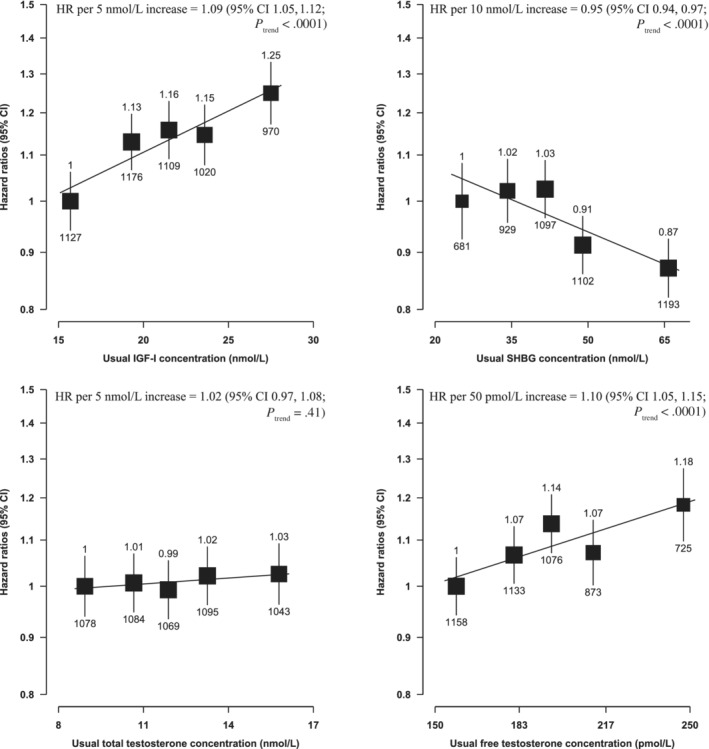
Hazard ratios of incident prostate cancer by fifths of usual serum hormone concentrations in UK Biobank. HRs are stratified by region (10 UK cancer registry regions) and age at recruitment (<45, 45‐49, 50‐54, 55‐59, 60‐64 and ≥65 years) and adjusted for age (underlying time variable), Townsend deprivation score (fifths, unknown), racial/ethnic group (white, mixed background, Asian, black, other unknown), height (<170, ≥170‐<175, ≥175‐<180, ≥180 cm, unknown), lives with a wife or partner (no, yes), BMI (<25, ≥25‐<30, ≥30‐<35, ≥35 kg/m^2^), cigarette smoking (never, former, light smoker, heavy smoker, current unknown and smoking status unknown), alcohol consumption (non‐drinkers, <1‐<10, ≥10‐<20, ≥20 g ethanol/day, unknown) and diabetes (no, yes and unknown). HRs for trend are adjusted for regression dilution bias. The boxes represent the HRs; the vertical lines represent the 95% CIs, with the size inversely proportional to the variance of the logarithm of the HR. The numbers above the vertical lines are point estimates for HRs, and the numbers below are the number of prostate cancer diagnoses. BMI, body mass index; CI, confidence intervals; HR, hazard ratio; IGF‐I, insulin‐like growth factor‐I; SHBG, sex hormone‐binding globulin

**FIGURE 2:**
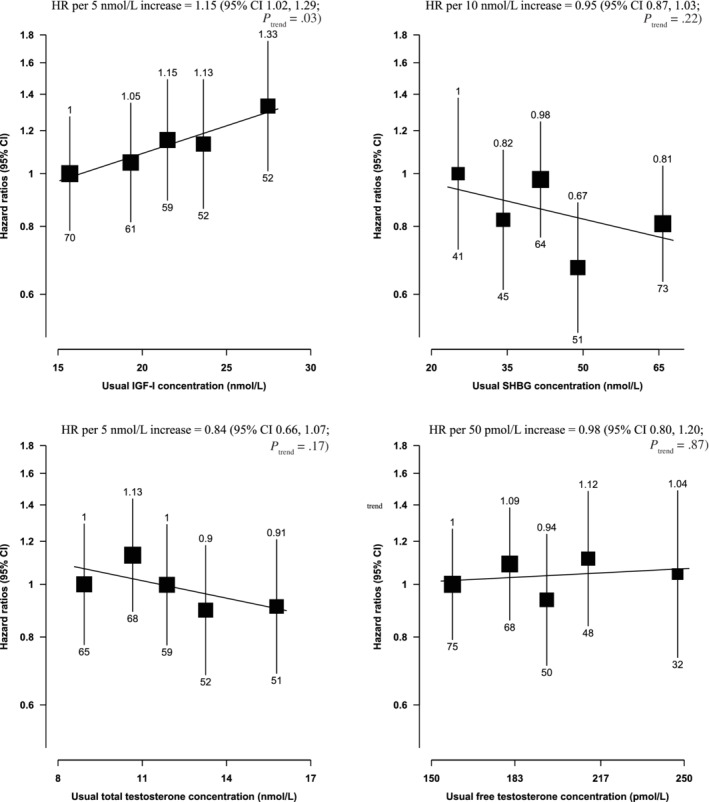
Hazard ratios of prostate cancer mortality by fifths of usual serum hormone concentrations in the UK Biobank. HRs are stratified by region (10 UK cancer registry regions) and age at recruitment (<45, 45‐49, 50‐54, 55‐59, 60‐64, and ≥65 years) and adjusted for age (underlying time variable), Townsend deprivation score (fifths, unknown), racial/ethnic group (white, mixed background, Asian, black, other, unknown), height (<170, ≥170‐<175, ≥175‐<180, ≥180 cm, unknown), lives with a wife or partner (no, yes), BMI (<25, ≥25‐<30, ≥30‐<35, ≥35 kg/m^2^), cigarette smoking (never, former, light smoker, heavy smoker, current unknown and smoking status unknown), alcohol consumption (non‐drinkers, <1‐<10, ≥10‐<20, ≥20 g ethanol/day, unknown) and diabetes (no, yes and unknown). HRs for trend are adjusted for regression dilution bias. The boxes represent the HRs; the vertical lines represent the 95% CIs, with the size inversely proportional to the variance of the logarithm of the HR. The numbers above the vertical lines are point estimates for HRs, and the numbers below are the number of prostate cancer deaths. BMI, body mass index; CI, confidence intervals; HR, hazard ratio; IGF‐I, insulin‐like growth factor‐I; SHBG, sex hormone‐binding globulin

Serum IGF‐I concentration was positively associated with prostate cancer incidence (HR per 5 nmol/L increment = 1.09, 95% CI 1.05‐1.12; *P*_trend_ < .0001, Figure 1) and prostate cancer mortality (HR per 5 nmol/L increment =1.15, 95% CI 1.02‐1.29; *P*_trend_ = .03, Figure 2).

Serum SHBG concentration was inversely associated with prostate cancer incidence (HR per 10 nmol/L increment = 0.95, 95% CI 0.94‐0.97; *P*_trend_ < .0001, Figure 1), but was not associated with prostate cancer mortality (Figure 2). Free testosterone was positively associated with prostate cancer incidence (HR per 50 pmol/L increment = 1.10, 95% CI 1.05‐1.15; *P*_trend_ < .0001, Figure 1) but not with mortality. Total testosterone was not associated with prostate cancer incidence or mortality.

Risk estimates with and without adjustment for regression dilution bias are shown in Supplementary Table S2.

#### 3.1.2 Subgroup analyses

**FIGURE 3:**
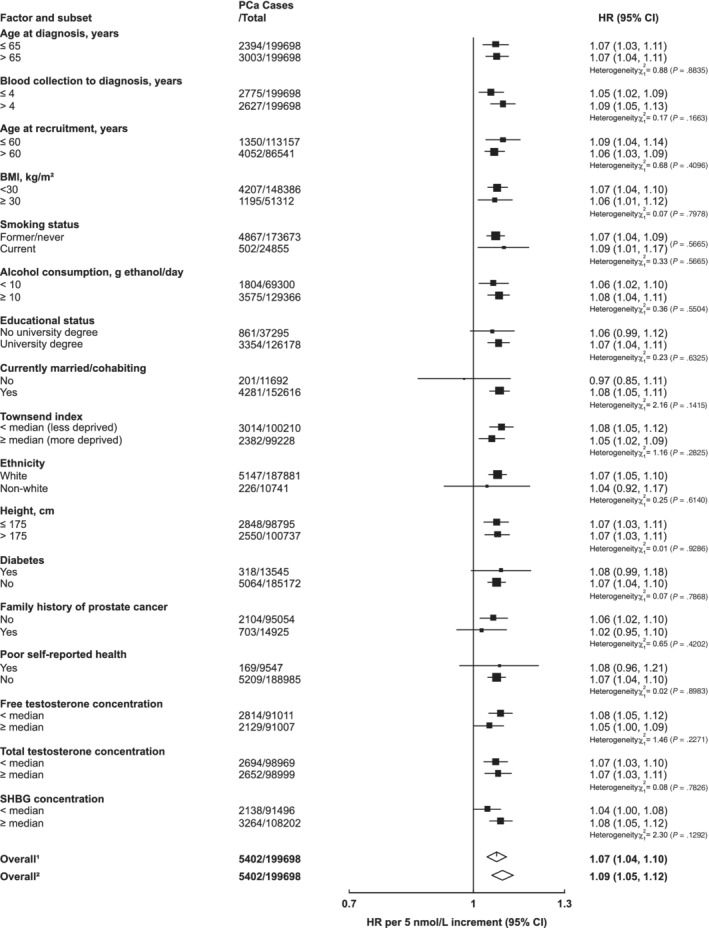
Hazard ratios of incident prostate cancer per 5 nmol/L increase in serum IGF‐I concentration by subgroup in the UK Biobank. Cox models based on competing risks and compared the risk coefficients and SEs in the two subgroups and tested using a *χ* ^2^ test of heterogeneity. For non‐case specific factors, heterogeneity was assessed using a *χ* ^2^ interaction term. HRs are stratified by region (10 UK cancer registry regions) and age at recruitment (<45, 45‐49, 50‐54, 55‐59, 60‐64, and ≥65 years) and adjusted for age (underlying time variable), Townsend deprivation score (fifths, unknown), racial/ethnic group (white, mixed background, Asian, black, other, unknown), height (<170, ≥170‐<175, ≥175‐<180, ≥180 cm, unknown), lives with a wife or partner (no, yes), BMI (<25, ≥25‐<30, ≥30‐<35, ≥35 kg/m^2^), cigarette smoking (never, former, light smoker, heavy smoker, current unknown and smoking status unknown), alcohol consumption (non‐drinkers, <1‐<10, ≥10‐<20, ≥20 g ethanol/day, unknown) and diabetes (no, yes and unknown). The boxes represent the HRs; the horizontal lines represent the 95% CIs, with the size inversely proportional to the variance of the logarithm of the HR. (1) Not adjusted for regression dilution bias. (2) Adjusted for regression dilution bias. BMI, body mass index; CI, confidence intervals; HR, hazard ratio; IGF‐I, insulin‐like growth factor‐I; PCa, prostate cancer

**FIGURE 4:**
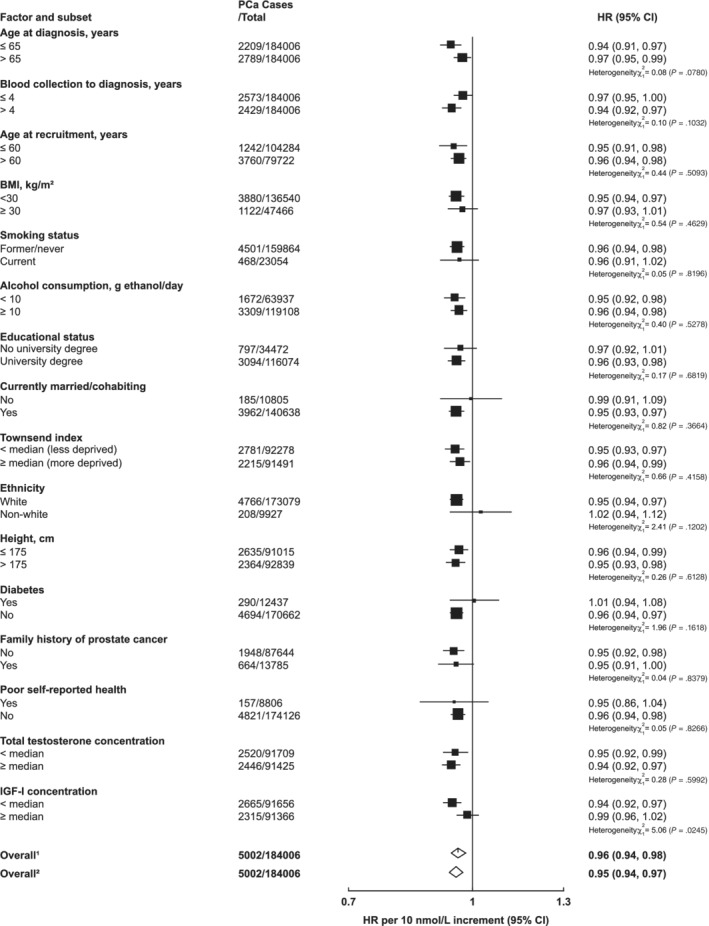
Hazard ratio of incident prostate cancer per 10 nmol/L increase in serum SHBG concentration by subgroup in the UK Biobank. Cox models based on competing risks and compared the risk coefficients and SEs in the two subgroups and tested using a *χ* ^2^ test of heterogeneity. For non‐case‐specific factors, heterogeneity was assessed using a *χ* ^2^ interaction term. HRs are stratified by region (10 UK cancer registry regions) and age at recruitment (<45, 45‐49, 50‐54, 55‐59, 60‐64 and ≥65 years) and adjusted for age (underlying time variable), and adjusted for Townsend deprivation score (fifths, unknown), racial/ethnic group (white, mixed background, Asian, black, other, unknown), height (<170, ≥170‐<175, ≥175‐<180, ≥180 cm, unknown), lives with a wife or partner (no, yes), BMI (<25, ≥25‐<30, ≥30‐<35, ≥35 kg/m^2^), cigarette smoking (never, former, light smoker, heavy smoker, current unknown and smoking status unknown), alcohol consumption (non‐drinkers, <1‐<10, ≥10‐<20, ≥20 g ethanol/day, unknown) and diabetes (no, yes and unknown). The boxes represent the HRs; the horizontal lines represent the 95% CIs, with the size inversely proportional to the variance of the logarithm of the HR. (1) Not adjusted for regression dilution bias. (2) Adjusted for regression dilution bias. BMI, body mass index; CI, confidence intervals; HR, hazard ratio; PCa, prostate cancer; SHBG, sex hormone‐binding globulin

There was no evidence of heterogeneity in the associations of IGF‐I with incident prostate cancer by any of the selected characteristics (Figure 3). There was some evidence that the inverse association between SHBG and prostate cancer incidence varied by IGF‐I concentration (*P*_het_ = .02); only men with lower concentrations of IGF‐I (< the study median) had a reduced risk of prostate cancer (HR per 10 nmol/L increment in SHBG = 0.94, 95% CI 0.92‐0.97, Figure 4).

**FIGURE 5:**
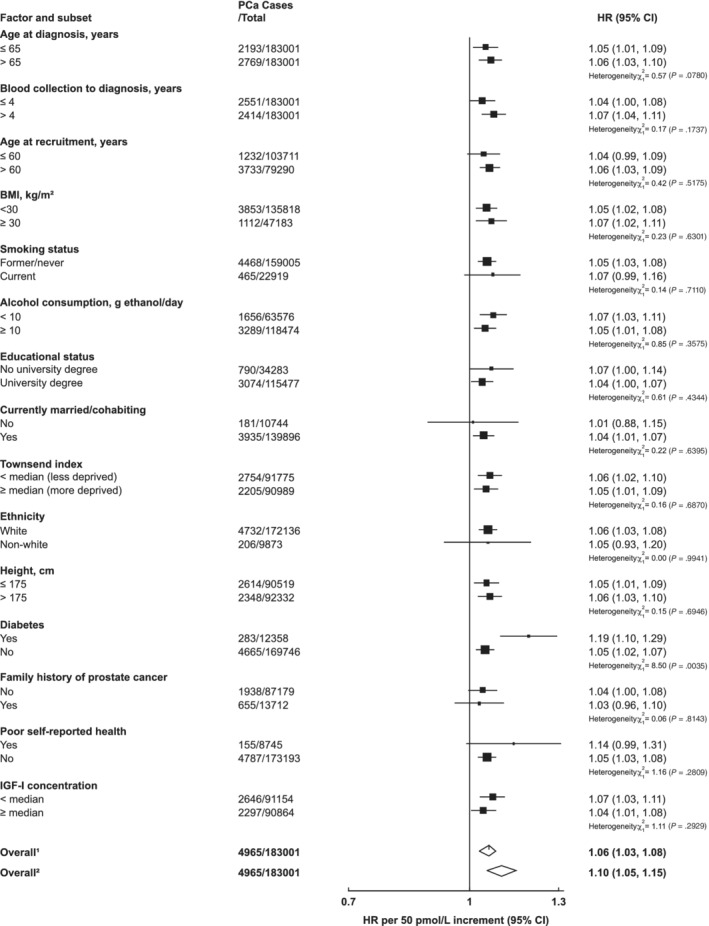
Hazard ratio of incident prostate cancer per 50 pmol/L increase in serum free testosterone concentration by subgroup in the UK Biobank. Cox models based on competing risks and compared the risk coefficients and SEs in the two subgroups and tested using a *χ* ^2^ test of heterogeneity. For non‐case‐specific factors, heterogeneity was assessed using a *χ* ^2^ interaction term. HRs are stratified by region (10 UK cancer registry regions) and age at recruitment (<45, 45‐49, 50‐54, 55‐59, 60‐64, and ≥65 years) and adjusted for age (underlying time variable), and adjusted for Townsend deprivation score (fifths, unknown), racial/ethnic group (white, mixed background, Asian, black, other, unknown), height (<170, ≥170‐<175, ≥175‐<180, ≥180 cm, unknown), lives with a wife or partner (no, yes), BMI (<25, ≥25‐<30, ≥30‐<35, ≥35 kg/m^2^), cigarette smoking (never, former, light smoker, heavy smoker, current unknown and smoking status unknown), alcohol consumption (non‐drinkers, <1‐<10, ≥10‐<20, ≥20 g ethanol/day, unknown) and diabetes (no, yes and unknown). The boxes represent the HRs; the horizontal lines represent the 95% CIs, with the size inversely proportional to the variance of the logarithm of the HR. (1) Not adjusted for regression dilution bias. (2) Adjusted for regression dilution bias. BMI, body mass index; CI, confidence intervals; HR, hazard ratio; PCa, prostate cancer

There was no evidence of heterogeneity in the associations of total or free testosterone with prostate cancer incidence by any characteristics except for diabetes status. For free testosterone, there was evidence that the magnitude of the association with incident prostate cancer was greater in men who were diabetic at baseline (HR per 50 pmol/L increment = 1.19, 95% CI 1.10‐1.29) than in those who were not (HR = 1.05, 95% CI 1.02‐1.07; *P*_het_ = .004, Figure 5). Total testosterone was also associated with prostate cancer in men with Type II diabetes, but not in men without diabetes (Supplementary Figure S2).

#### 3.1.3 Further analyses

Associations with incident prostate cancer remained broadly similar when the associations were examined across tenths of the distributions (Supplementary Figure S1), and per 80 percentile increase (Supplementary Table S3). Minimally adjusted results are displayed in Supplementary Table S4. Mutual adjustment for hormones did not materially affect the risk estimates (Supplementary Table S4).

### 3.2 Mendelian randomisation

MR analysis using the *cis*‐SNP found that IGF‐I was significantly associated with a 34% increased prostate cancer risk per 5 nmol/L increment (95% CI 1.07‐1.68; *P* = .01) (Table 2; Supplementary Figure S3).

**TABLE 2:** Mendelian randomisation estimates between genetically predicted circulating IGF‐I concentrations and prostate cancer risk

	Method	OR per genetically predicted 5 nmol/L increase in IGF‐I (95% CI)	*P* value
*cis*‐SNP (*N* = 1)	Wald ratio	1.34 (1.07–1.68)	.01
All SNPs (*trans*‐ and *cis‐*SNPs, *N* = 122)	Inverse weighted variance	1.06 (1.00–1.13)	.06
	Weighted median	1.03 (0.96‐1.11)	.43
	Weighted mode	0.99 (0.87‐1.11)	.82
	MR‐Egger	1.04 (0.90‐1.20)	.57
	MR‐PRESSO	1.06 (1.00‐1.12)	.04

Abbreviations: CI, confidence interval; IGF‐I, insulin‐like growth factor‐I; OR, odds ratio; SNP, single nucleotide polymorphism.

MR analysis including all the SNPs (both the *cis* and *trans*‐SNPs) found suggestive evidence between IGF‐I and prostate cancer diagnosis in the same direction as the *cis*‐SNP results (inverse‐variance weighted OR for a genetically predicted 5 nmol/L increment in IGF‐I = 1.06, 95% CI 1.00‐1.13; *P* = .06, Table 2). Although the Egger intercept did not indicate the presence of directional pleiotropy, significant heterogeneity (Cochran's *Q P* < .0001) may have influenced SE estimation for the inverse‐variance weighted estimate (Table 2). Results were consistent following the removal of outliers identified using the MR‐PRESSO outlier test (Table 2), and leave‐one‐out analyses, including removal of the *cis*‐SNP (data not shown).

There is no direct functional evidence for the *cis*‐SNP. However, this SNP is in modest linkage disequilibrium with the SNPs rs17727841, rs10860864, rs5742671, rs1996656 and rs11111250, which are expression quantitative trait loci (eQTLs) associated with *IGF1* and *WASHC3* expression.^36^ PheWAS using published data showed the *cis*‐SNP was associated with measures of lung function and adiposity (Supplementary Table S5). There was a large amount of pleiotropy in the *trans* genetic instruments, for example, for the top 100 *trans*‐SNPs most strongly associated with IGF‐I, there were >2500 published genome‐wide significant associations (using the PhenoScanner resource).

## 4 DISCUSSION

Our observational and MR analyses provide strong evidence that men with higher circulating IGF‐I have an elevated risk of prostate cancer; furthermore, our observational analyses suggest a higher risk of prostate cancer mortality in these men, suggesting that IGF‐I is associated with risk for more severe forms of prostate cancer and/or may increase the risk of prostate cancer progression. Higher serum‐free testosterone was associated with a higher risk of prostate cancer diagnosis, which is supported by a recent MR analysis.^9^ We also found that men with a higher SHBG had a lower risk. Total testosterone concentration was not associated with prostate cancer incidence or mortality.

The findings of a likely causal effect of IGF‐I in prostate cancer development may be due to its role in activating signalling pathways, which regulate cell proliferation and apoptosis.^2^ The positive relationship between IGF‐I and incident prostate cancer observed is consistent with previous epidemiological evidence,^4^ as well as associations observed with other cancers including breast and colorectal.^37^, ^38^, ^39^ Further genetic epidemiology including fine mapping may help elucidate exactly by which mechanism variation at the *IGF1* locus associates with risk of prostate and some other cancers.

Our finding of a positive association between calculated free testosterone concentration and prostate cancer diagnosis is consistent with the importance of androgens for prostate cancer development,^3^ previous evidence from pooled nested case–control studies,^8^ randomised controlled trials that aim to reduce intraprostatic androgen signalling^40^, ^41^ and MR analyses.^9^ However, the shape of the association between circulating free testosterone and prostate cancer risk is not completely clear; here we see an approximately linear association, whereas our previous pooled analysis suggested that very low free testosterone concentrations were associated with a lower prostate cancer risk, but that risk did not change with further increments in free testosterone concentration.^8^

There is some evidence to suggest that men with low free testosterone may have an increased risk of high‐grade prostate tumours, but the data are inconclusive.^8^, ^40^, ^41^, ^42^ In this analysis, prostate cancer mortality was used as a proxy of tumour aggressiveness; we did not observe an association between circulating total or free testosterone concentrations and prostate cancer mortality, although statistical power to examine this association was limited (<300 prostate cancer deaths).

Men with higher SHBG concentrations had a lower risk of prostate cancer, which is consistent with previous prospective studies.^8^ MR analyses have also shown some evidence of an inverse relationship, but results were not statistically significant.^9^ In the current study, we cannot determine whether the mechanism underlying this association relates to SHBG itself or to the action of SHBG as a carrier protein, which modulates androgen access to tissues.

This analysis has several strengths. It is the largest prospective full‐cohort analysis to examine hormones in relation to prostate cancer incidence and mortality. UK Biobank is a well‐characterised study population, and therefore we were able to adjust our risk results for a wide range of possible confounders and to investigate associations in a number of subgroups. Our results were consistent across these subgroups; in only three subgroup analyses, there was weak evidence of heterogeneity in the associations with prostate cancer risk. We observed heterogeneity in the associations of total and free testosterone by diabetes status. This heterogeneity may be related to hormone differences in diabetic men, who have a lower risk of prostate cancer diagnosis.^21^ However, men with diabetes also have lower PSA concentrations, which reduces the probability of prostate cancer detection^43^, ^44^ and may lead to differential bias of our risk estimates; we are also unable to rule‐out chance due to multiple testing and the small numbers of men with diabetes and subsequent prostate cancer (n = 283‐311). Hormones were measured using a standardised method; therefore, we were able to estimate risk associations of the absolute scale and use repeat measurements to improve the precision of risk estimates.^22^ Furthermore, by incorporating both observational and MR methods, we were able to use different lines of evidence with orthogonal biases to investigate the potential causality of the associations of IGF‐I with prostate cancer risk.^45^ Our MR analysis of IGF‐I using a *cis*‐SNP is an example of the strongest case for an MR analysis, due to the strong plausibility of a biological link and a reduced likelihood of horizontal pleiotropy^46^, ^47^; therefore the association for this *cis‐*SNP indicates that IGF‐I may be driving the reported associations with prostate cancer risk.

A limitation of the analysis is that prostate tumour stage and grade information are not currently available in the UK Biobank, and only incident prostate cancer data are available in the MR analyses. The UK Biobank participants are predominantly white and healthier than the sampling population; therefore, selection bias may influence the results^48^ and risk estimates may not be generalisable,^13^ although this is unlikely to affect the direction of the associations.^49^ Relatively weak evidence from MR analyses incorporating all GWAS significant SNPs for IGF‐I may reflect widespread pleiotropy for *trans*‐SNPs including the IGFBPs and IGF‐II signalling, which served as primary motivation to emphasise the association of the *cis*‐variant. A limitation of the use of this *cis* instrument is the lack of direct functional evidence; however, the identification of an individual SNP linked to an eQTL is difficult because of the high collinearity of SNPs. There was some evidence that SNPs in modest linkage disequilibrium with the *cis*‐variant alter the expression levels of *IGF1* and *WASHC3*. Due to the strong association of rs5742653 with circulating IGF‐I concentrations, it's location within the *IGF1* gene region, and the lack of any other biological evidence linking *WASHC3* to prostate cancer risk, we consider that the use of this *cis*‐SNP is valid. The effect estimates from the MR analyses were calculated on the same scale as for the observational analyses, and this scaling‐up results in some imprecision with wide confidence intervals in the association with the *cis*‐SNP; the concordance of the directions of the associations is particularly important.

Testosterone is related to other androgens, which have not been measured in UK Biobank; therefore, associations may be at least partially be explained by other androgens, although there is little observational evidence to support this.^50^ Free testosterone concentrations were estimated using a commonly employed formula derived from mass action equations, and the results may not correspond precisely to free testosterone measured using equilibrium dialysis.^51^ SHBG circulates as a homodimer, which presents two binding sites for testosterone adding further complexity to calculation of free testosterone.^7^ Furthermore, experimental evidence postulates entry of SHBG‐bound testosterone into cells via megalin, an endocytic receptor expressed in reproductive tissues.^52^ Therefore, the predictive value of calculated free testosterone as an indicator of the bioavailability of testosterone to the cells within the prostate remains under debate.^53^ Measurement of serum‐free testosterone using equilibrium dialysis may improve the accuracy of risk estimates, but this methodology is labour‐intensive and not readily available.

PSA concentrations are partly regulated by the androgen receptor^54^; lower free testosterone concentrations may therefore reduce circulating PSA concentrations, reducing the likelihood of prostate cancer detection (possibly as well as reducing the likelihood of cancer development). Comorbidities, socioeconomic status and poor health may affect PSA test attendance, but PSA testing attendance after baseline was not known.

In conclusion, our results implicate IGF‐I and free testosterone in prostate cancer development and/or progression. This analysis of 200 000 men enabled us to quantify the associations of circulating hormone concentrations with prostate cancer risk. The complementary MR for IGF‐I supports a causal association. Future research will examine hormone associations by tumour stage and grade.

## DISCLAIMERS

Department of Health and Social Care disclaimer: The views expressed are those of the author(s) and not necessarily those of the NHS, the NIHR or the Department of Health and Social Care.

Where authors are identified as personnel of the International Agency for Research on Cancer/World Health Organization, the authors alone are responsible for the views expressed in this article and they do not necessarily represent the decisions, policy or views of the International Agency for Research on Cancer/World Health Organization.

## CONFLICT OF INTEREST

Dr Holmes has collaborated with Boehringer Ingelheim in research, and in adherence to the University of Oxford's Clinical Trial Service Unit & Epidemiological Studies Unit (CSTU) staff policy, did not accept personal honoraria or other payments from pharmaceutical companies. All other authors have no competing interests to declare.

## ETHICS STATEMENT

The UK Biobank study was approved by the North West Multi‐Centre Research Ethics Committee (reference number 06/MRE08/65) and at recruitment all participants gave written informed consent to participate and for their health to be followed‐up through linkage to electronic medical records.

## Supporting information


**Appendix** **S1:** Supporting information

## Data Availability

UK Biobank is an open access resource, and the study website https://www.ukbiobank.ac.uk/ has information on available data and access procedures. GWAS results are publicly available from http://www.nealelab.is/uk-biobank. PRACTICAL data are available upon application via the consortium website http://practical.icr.ac.uk/blog/. Additional datasets used in the analyses will be made available upon reasonable request.
