# Supplementary material for: Circulating insulin‐like growth factor‐I, total and free testosterone concentrations and prostate cancer risk in 200 000 men in UK Biobank
Source: Int J Cancer. 2020 Dec 11;148(9):2274–88. doi: 10.1002/ijc.33416 (PMC8048461; doi:10.1002/ijc.33416)
Supplement: Supplementary file 1 — Appendix S1: Supporting information [file IJC-148-2274-s001.pdf]

Supplementary Material

Circulating insulin-like growth factor-I, total and free testosterone concentrations and prostate cancer risk in 200,000 men in UK Biobank

Eleanor L. Watts, Georgina K. Fensom, Karl Smith Byrne, Aurora Perez-Cornago, Naomi E. Allen, Anika Knuppel, Marc J. Gunter, Michael V. Holmes, Richard M. Martin, Neil Murphy, Konstantinos K. Tsilidis, Bu B. Yeap, Timothy J. Key, Ruth C. Travis

Table of contents

Table S1: Association of IGF-I SNPs used in 2-sample Mendelian randomization analyses…..……………………………………………………………………...…….Page 2

Table S2: Associations of serum biomarker concentrations with prostate cancer incidence and mortality in UK Biobank, with and without correction for regression dilution bias……………………………………………………………………………………….Page 6

Table S3: Associations of biomarkers with prostate cancer incidence and mortality per 80% increase in concentration†……………………..…………………………..…….Page 7

Table S4: Associations of biomarkers with prostate cancer incidence with additional adjustment for other hormone concentrations…………………………………..…….Page 8

Table S5: PheWAS associations of the IGF-I *cis*-SNP...……………………………Page 9

Figure S1: Hazard ratio of incident prostate cancer by tenths of serum hormone concentrations in UK Biobank……………………………………………….……………………Page 10

Figure S2: Hazard ratio of incident prostate cancer per 5 nmol/L increase in serum total testosterone concentrations by subgroup in the UK Biobank…………………………..Page 11

Figure S3: Scatterplot of genetic associations with IGF-I against genetic associations with prostate cancer risk………………………………………………………………..Page 12

Supplementary Table S1- Association of IGF-I SNPs used in 2-sample Mendelian randomization analyses

						Association parameters with IGF-I concentration			Association parameters with prostate cancer		
SNP	Chr	Position	Nearest gene	Effect allele	Other allele	Effect	SE	P-value	Effect	SE	P-value
rs17037452	1	11895675	CLCN6	A	G	0.143	0.026	3.45E-08	0.011	0.011	0.341
rs36086195	1	16510894	ARHGEF19-AS1:ARHGEF19	T	C	-0.128	0.019	4.33E-11	-0.007	0.008	0.389
rs142840389	1	29036445	GMEB1	A	G	0.167	0.029	1.36E-08	-0.004	0.013	0.744
rs12035012	1	41750648	RP11-399E6.1	A	C	-0.144	0.023	4.71E-10	-0.010	0.010	0.297
rs2842189	1	44007648	PTPRF	T	C	0.123	0.020	7.14E-10	0.016	0.008	0.050
rs56958645	1	65223626	RAVER2	T	C	0.201	0.032	1.95E-10	0.004	0.013	0.781
rs165316	1	91533297	RPL5P6	A	G	-0.446	0.024	4.43E-76	-0.021	0.010	0.043
rs12727665	1	92780085	RPAP2	T	G	0.150	0.023	1.12E-10	-0.009	0.010	0.354
rs599839	1	109822166	PSRC1	A	G	-0.178	0.023	5.96E-15	0.003	0.010	0.792
rs12749024	1	176522365	PAPPA2	T	C	0.374	0.027	2.74E-43	-0.013	0.012	0.274
rs1204706	1	208021060	C1orf132	T	G	0.125	0.020	1.91E-10	0.001	0.008	0.937
rs17764516	1	214224428	PROX1	T	C	0.155	0.029	7.67E-08	0.012	0.014	0.401
rs17597619	1	221047861	HLA-AS1	T	C	-0.265	0.022	1.73E-32	-0.008	0.010	0.393
rs12048930	1	243940076	AKT3	T	C	-0.200	0.025	4.07E-16	-0.018	0.010	0.071
rs35135518	2	16120506	AC010145.4	T	C	0.186	0.031	1.60E-09	0.010	0.015	0.514
rs1260326	2	27730940	GCKR	T	C	-0.292	0.020	4.77E-50	0.022	0.008	0.007
rs10169411	2	42637749	COX7A2L	A	G	-0.172	0.028	1.48E-09	-0.011	0.012	0.367
rs35624252	2	70282710	PCBP1-AS1	T	G	0.395	0.067	4.83E-09	0.016	0.034	0.633
rs17400325	2	178565913	PDE11A:AC012499.1	T	C	-0.276	0.048	1.21E-08	-0.038	0.021	0.065
rs1465529	2	231039037	SP110	T	C	0.115	0.021	4.01E-08	0.001	0.009	0.901
rs9847027	3	23319579	UBE2E2	T	G	-0.114	0.020	1.33E-08	-0.002	0.009	0.807
rs1716975	3	41960006	ULK4	T	C	-0.149	0.026	1.06E-08	-0.004	0.011	0.704
rs9824926	3	51763293	GRM2	T	G	-0.112	0.020	1.70E-08	-0.003	0.009	0.710
rs6551280	3	88201739	C3orf38	T	C	-0.147	0.027	3.75E-08	0.031	0.011	0.005
rs13088318	3	101242751	FAM172BP	A	G	-0.218	0.020	9.60E-27	-0.025	0.009	0.003
rs895893	3	135955604	PCCB	T	C	0.189	0.023	7.02E-17	0.012	0.010	0.227
rs55717031	3	138848505	MRPS22	T	G	-0.249	0.021	1.33E-32	0.010	0.009	0.278
rs73238159	3	142078759	XRN1	T	C	-0.186	0.029	8.07E-11	-0.024	0.012	0.046
rs13073970	3	170630520	EIF5A2	T	G	0.138	0.024	6.63E-09	-0.015	0.011	0.153
rs572169	3	172165727	GHSR	T	C	0.307	0.021	1.87E-50	0.017	0.009	0.053
rs4689088	4	7222253	SORCS2	A	G	0.169	0.020	1.43E-17	-0.003	0.008	0.712
rs1398263	4	45122349	RP11-362I1.1	T	C	0.211	0.020	3.35E-25	-0.013	0.009	0.131
rs9884390	4	69373407	UGT2B29P	T	C	0.282	0.023	5.19E-35	0.003	0.011	0.770
rs139916529	4	69564066	RP11-1267H10.4	T	G	0.220	0.038	5.77E-09	-0.034	0.018	0.053
rs111369964	4	90010362	FAM13A	T	C	0.163	0.025	8.01E-11	0.036	0.010	0.001
rs1229984	4	100239319	ADH1B	T	C	0.807	0.065	7.69E-36	-0.035	0.023	0.127
rs12641157	4	148987295	ARHGAP10	T	G	-0.172	0.022	1.68E-14	-0.012	0.010	0.215
rs55681913	5	42687629	GHR	T	C	-0.321	0.031	1.57E-24	0.005	0.014	0.749
rs315262	5	42888022	SEPP1	T	C	0.149	0.022	4.72E-12	0.011	0.010	0.229
rs7719168	5	53292390	ARL15	A	C	-0.173	0.030	1.49E-08	0.002	0.013	0.860
rs11738977	5	59018442	PDE4D	A	G	0.180	0.021	2.04E-18	-0.011	0.009	0.195
rs9293511	5	88416354	MEF2C-AS1	T	C	-0.168	0.020	2.02E-17	0.002	0.009	0.813
rs12514133	5	137742465	KDM3B	T	C	-0.206	0.024	7.85E-18	-0.025	0.010	0.012
rs2974433	5	168268387	SLIT3	T	C	0.265	0.023	9.89E-32	-0.004	0.010	0.689
rs9379822	6	26070672	HIST1H1C	T	G	0.225	0.020	2.85E-28	0.018	0.008	0.033
rs9470825	6	38162310	BTBD9	A	G	-0.154	0.022	2.50E-12	0.002	0.009	0.857
rs395962	6	105397418	LIN28B	T	G	-0.218	0.021	2.78E-26	-0.008	0.009	0.348
rs2153960	6	108988184	FOXO3	A	G	0.269	0.021	4.86E-37	0.012	0.009	0.177
rs790513	6	154420368	OPRM1	A	C	-0.192	0.022	4.20E-18	0.003	0.009	0.739
rs7381453	6	166313164	PDE10A	A	G	-0.254	0.020	7.61E-38	-0.023	0.009	0.007
rs7802508	7	1191689	ZFAND2A	A	G	0.148	0.019	3.75E-14	-0.004	0.008	0.649
rs12702534	7	6733945	AC073343.13:ZNF12	A	G	0.290	0.028	1.76E-25	0.002	0.012	0.859
rs112293610	7	14226261	DGKB	A	C	0.141	0.021	3.19E-11	-0.013	0.009	0.161
rs4988501	7	31011485	GHRHR	A	G	0.168	0.022	2.21E-14	-0.010	0.011	0.368
rs62460538	7	44940256	RP4-673M15.1	T	C	0.249	0.038	3.29E-11	0.021	0.017	0.221
rs10243669	7	45915907	AC096582.1	T	C	-0.219	0.027	6.99E-16	0.005	0.011	0.653
rs700750	7	46753491	AC011294.3	A	C	0.618	0.020	1.00E-200	-0.004	0.008	0.622
rs10262462	7	114180062	FOXP2	A	G	0.126	0.020	2.07E-10	0.008	0.008	0.344
rs157934	7	130585492	AC016831.7:MIR29A	T	C	-0.326	0.021	5.68E-55	0.010	0.009	0.275

rs41341748	8	16012594	MSR1	A	G	-0.564	0.091	4.74E-10	0.004	0.041	0.924
rs4738684	8	59393273	CYP7A1	A	G	0.181	0.020	6.21E-19	0.001	0.009	0.906
rs7010636	8	77081903	RNU2-54P	A	G	-0.130	0.020	2.83E-11	0.010	0.008	0.203
rs7010330	8	145036615	PLEC	T	C	-0.108	0.020	4.46E-08	-0.008	0.008	0.350
rs295263	9	4840063	RCL1	T	C	0.223	0.030	4.92E-14	0.024	0.013	0.066
rs41303235	9	4985388	JAK2	T	C	0.336	0.047	7.88E-13	-0.036	0.022	0.098
rs62560861	9	34078582	RP11-537H15.3	A	C	0.142	0.024	5.67E-09	-0.029	0.010	0.005
rs10869022	9	74057313	TRPM3	T	C	-0.176	0.024	3.19E-13	-0.023	0.010	0.031
rs10156602	9	96345328	PHF2	A	G	0.151	0.020	5.55E-14	0.004	0.008	0.665
rs925813	9	97525627	C9orf3	T	C	0.288	0.039	3.17E-13	0.030	0.017	0.067
rs78509281	9	109566543	RNA5SP292	T	C	0.294	0.044	3.66E-11	-0.029	0.019	0.129
rs13302549	9	128284775	MAPKAP1	T	C	0.123	0.020	5.97E-10	-0.016	0.009	0.064
rs1832007	10	5254847	AKR1C4	A	G	-0.205	0.027	1.18E-14	0.016	0.011	0.141
rs10821719	10	62076964	ANK3	T	C	-0.111	0.020	2.07E-08	0.000	0.008	0.992
rs3842763	11	2179204	INS-IGF2	T	G	-0.412	0.023	1.09E-72	-0.009	0.009	0.335
rs1037169	11	13361005	ARNTL	T	C	-0.123	0.021	3.01E-09	-0.012	0.009	0.163
rs1039481	11	48182237	PTPRJ	A	G	-0.227	0.022	3.00E-25	0.006	0.009	0.536
rs174564	11	61588305	FADS2:FADS1	A	G	0.178	0.020	8.24E-19	0.002	0.009	0.864
rs117104648	11	65543736	AP5B1	T	C	-0.235	0.040	5.09E-09	0.026	0.017	0.133
rs71477696	11	94133404	GPR83	T	G	0.418	0.054	8.98E-15	0.026	0.027	0.334
rs2856321	12	11855773	ETV6	A	G	-0.115	0.020	1.01E-08	-0.014	0.008	0.103
rs9738365	12	31997635	RP11-428G5.4	A	C	0.359	0.022	4.34E-61	0.001	0.009	0.930
rs7953987	12	98167068	RP11-1016B18.1	A	G	0.151	0.020	2.29E-14	-0.001	0.008	0.892
rs5742653	12	102835859	IGF1	T	C	-0.390	0.022	5.38E-71	-0.023	0.009	0.010
rs1800574	12	121416864	HNF1A-AS1:HNF1A	T	C	0.925	0.057	4.29E-59	-0.010	0.025	0.671
rs2243582	13	21305153	N6AMT2	A	G	0.117	0.021	1.65E-08	0.006	0.009	0.510
rs10507482	13	40755641	LINC00332	A	G	-0.251	0.025	7.30E-24	0.008	0.011	0.447
rs1886220	13	47159484	LRCH1	A	G	-0.140	0.022	3.20E-10	-0.005	0.010	0.628
rs61957204	13	74084684	LINC00393	A	G	0.259	0.036	2.89E-13	-0.117	0.018	0.000
rs6602912	13	114546549	GAS6	T	G	-0.120	0.021	1.75E-08	-0.011	0.010	0.270
rs33912345	14	60976537	C14orf39:SIX6	A	C	-0.133	0.020	1.55E-11	0.010	0.008	0.243
rs10145740	14	74234294	ELMSAN1	T	C	-0.151	0.022	1.66E-11	-0.005	0.010	0.592
rs28929474	14	94844947	SERPINA1	T	C	-0.625	0.068	6.43E-20	-0.134	0.030	0.000
rs190543502	15	43757184	TP53BP1	T	C	0.759	0.064	1.43E-32	0.048	0.031	0.124
rs4777035	15	68605169	ITGA11	A	G	0.111	0.020	1.54E-08	-0.011	0.009	0.211
rs76819935	16	1110581	SSTR5-AS1	T	C	0.808	0.043	1.19E-79	0.007	0.025	0.781
rs8048693	16	1811565	MAPK8IP3	A	G	0.252	0.020	7.93E-36	0.024	0.009	0.005
rs1369924	16	5924291	RP11-420N3.2	A	C	0.207	0.025	4.22E-17	-0.016	0.011	0.132
rs73530203	16	31099859	PRSS53:RP11-196G11.1	A	G	-0.192	0.020	3.18E-22	-0.006	0.008	0.483
rs8062941	16	69578482	NFAT5	A	G	-0.177	0.027	3.46E-11	0.020	0.012	0.082
rs8059803	16	81603001	CMIP	A	G	0.212	0.021	5.03E-24	0.001	0.010	0.934
rs12950335	17	38215314	MED24:THRA	A	G	-0.108	0.020	3.84E-08	-0.015	0.008	0.059
rs1991556	17	44083402	MAPT	A	G	0.187	0.023	4.86E-16	-0.026	0.010	0.010
rs8070132	17	57790206	VMP1	T	C	0.155	0.027	7.48E-09	0.004	0.011	0.702
rs2005172	17	61996255	CSHL1	A	C	-0.205	0.020	3.81E-24	0.020	0.009	0.021
rs11658385	17	73810400	UNK	T	C	0.138	0.020	3.75E-12	0.006	0.009	0.501
rs4075482	17	79074548	BAIAP2	A	C	-0.119	0.020	2.53E-09	0.011	0.009	0.236
rs12454712	18	60845884	BCL2	T	C	0.126	0.020	2.50E-10	0.001	0.008	0.887
rs74327681	18	74981730	GALR1	T	C	-0.395	0.047	6.86E-17	0.004	0.021	0.832
rs12459464	19	5013831	KDM4B	T	C	0.197	0.021	1.46E-21	0.014	0.009	0.112
rs8105174	19	10347032	DNMT1	T	C	-0.275	0.025	4.41E-29	0.007	0.011	0.553
rs6510177	19	31211647	ZNF536	T	C	0.181	0.025	1.82E-13	-0.015	0.013	0.226
rs12975366	19	54759361	LILRB5	T	C	0.117	0.020	2.42E-09	0.011	0.009	0.209
rs73125628	20	20066701	C20orf26	T	C	-0.239	0.021	8.72E-29	0.013	0.009	0.141
rs1072271	20	21151377	PLK1S1:RP4-777D9.2	A	G	-0.328	0.020	6.37E-58	-0.017	0.009	0.048
rs2268879	20	25177805	ENTPD6	T	C	0.108	0.020	3.95E-08	0.020	0.008	0.016
rs17265513	20	39832628	ZHX3	T	C	0.188	0.024	4.67E-15	0.014	0.010	0.193
rs932792	20	54851746	RP11-380D15.2	A	C	-0.151	0.020	6.28E-14	-0.005	0.008	0.523
rs185799410	20	57466093	GNAS	T	G	-0.365	0.062	2.83E-09	-0.014	0.029	0.622
rs17274750	21	16353809	NRIP1:AF127577.11	A	C	0.202	0.033	5.16E-10	-0.003	0.014	0.817
rs7280982	21	37468223	AP000688.14	A	G	-0.284	0.023	1.94E-34	-0.028	0.010	0.003
rs6519133	22	39096602	JOSD1	T	C	0.139	0.020	1.44E-12	-0.002	0.008	0.830
rs16991158	22	44327179	PNPLA3	A	G	-0.181	0.026	6.25E-12	-0.030	0.011	0.005

Abbreviations: Chr=chromosome; SE=standard error.

Supplementary Table S2: Associations of serum biomarker concentrations with prostate cancer incidence and mortality in UK Biobank, with and without correction for regression dilution bias

Biomarker	N*	Mean concentration at baseline (SD)*	Mean concentration at resurvey (SD)*	MacMahon-Peto regression ratio†	Intra-class correlation coefficients	Incidence		Mortality	
						Uncorrected for RDB	Corrected for RDB	Uncorrected for RDB	Corrected for RDB
HR per 5 nmol/L increase in IGF-I (95% CI)	7776	22.05(5.22)	21.60(5.37)	0.80	0.78	1.07 (1.04-1.10)	1.09 (1.05-1.12)	1.12 (1.01-1.23)	1.15 (1.02-1.29)
HR per 10 nmol/L increase in SHBG (95% CI)	6387	40.24(16.31)	43.98(17.65)	0.93	0.82	0.96 (0.94-0.98)	0.95 (0.94-0.97)	0.95 (0.88-1.03)	0.95 (0.87-1.03)
HR per 5 nmol/L increase in total testosterone (95% CI)	7694	12.11(3.53)	12.19(3.66)	0.70	0.66	1.02 (0.98-1.06)	1.02 (0.97-1.08)	0.89 (0.75-1.05)	0.84 (0.66-1.07)
HR per 50 pmol/L increase in free testosterone (95% CI)	6285	209.46(57.39)	199.18(60.53)	0.57	0.54	1.06 (1.03-1.08)	1.10 (1.05-1.15)	0.99 (0.88-1.11)	0.98 (0.80-1.20)

HRs are stratified by region (10 UK cancer registry regions) and age at recruitment (<45, 45–49, 50–54, 55–59, 60–64, and ≥65 years) and adjusted for age (underlying time variable), and adjusted for Townsend deprivation score (fifths, unknown), racial/ethnic group (white, mixed background, Asian, black, other, unknown) , height (<170, ≥170–<175, ≥175–<180, ≥180 cm, unknown), lives with a wife or partner (no, yes), BMI (<25, ≥25–<30, ≥30–<35, ≥35 kg/m^2), cigarette smoking (never, former, light smoker, heavy smoker, current unknown, and smoking status unknown), alcohol consumption (non-drinkers, <1-<10, ≥10-<20, ≥20 g ethanol/day, unknown), and diabetes (no, yes, and unknown).

*Values are restricted to men with both baseline and repeat measurements.

†R_r/R_b where:

R_r=difference between the mean value at resurvey in the highest fifth of the distribution at baseline and mean value at resurvey in the lowest fifth of the distribution at baseline.

R_b= difference between the mean value in the highest fifth of the distribution at baseline and the mean value in the lowest fifth of the distribution at baseline.

Abbreviations: BMI=body mass index; CI=confidence interval; HR=hazard ratio; IGF-I=insulin-like growth factor-I; RDB=regression dilution bias; SD=standard deviation; SHBG=sex hormone binding globulin.

Supplementary Table S3: Associations of biomarkers with prostate cancer incidence and mortality **per 80% increase in concentration**†

Biomarker	Incidence, HR (95% CI)*	Mortality, HR (95% CI)*
IGF-I (nmol/L)	1.26 (1.14-1.38)	1.38 (0.91-2.08)
SHBG (nmol/L)	0.83 (0.76-0.91)	0.78 (0.52-1.16)
Total testosterone (nmol/L)	1.04 (0.93-1.16)	0.79 (0.48-1.29)
Free testosterone (pmol/L)	1.27 (1.10-1.47)	1.08 (0.57-2.03)

*HRs per 80% increase in biomarker concentrations are stratified by region (10 UK cancer registry regions) and age at recruitment (<45, 45–49, 50–54, 55–59, 60–64, and ≥65 years) and adjusted for age (underlying time variable), and adjusted for Townsend deprivation score (fifths, unknown), racial/ethnic group (white, mixed background, Asian, black, other, unknown) , height (<170, ≥170–<175, ≥175–<180, ≥180 cm, unknown), lives with a wife or partner (no, yes), BMI (<25, ≥25–<30, ≥30–<35, ≥35 kg/m^2), cigarette smoking (never, former, light smoker, heavy smoker, current unknown, and smoking status unknown), alcohol consumption (non-drinkers, <1-<10, ≥10-<20, ≥20 g ethanol/day, unknown), and diabetes (no, yes, and unknown). Risk estimates are adjusted for RDB.
† risk estimates were calculated using fifths of the distributions scored as 0, 0.25, 0.5, 0.75, and 1 and entered into the model as a continuous variable such that the mid-points of the lowest and highest fifths are the 10th and 90th percentiles of the hormones concentration. Therefore, a unit increase in this variable can be taken to represent an 80th percentile increase in the hormone concentrations.
Abbreviations: BMI=body mass index; CI=confidence interval; HR=hazard ratio; IGF-I=insulin-like growth factor-I; RDB=regression dilution bias; SHBG=sex hormone binding globulin

Supplementary Table S4: Associations of biomarkers with prostate cancer incidence with additional adjustment for other hormone concentrations

Biomarker		Fifths					Per x unit increase	
		1. HR(95% CI)	2. HR(95% CI)	3. HR(95% CI)	4. HR(95% CI)	5. HR(95% CI)	HR (95% CI)	P-value
IGF-I (per 5 nmol/L increase)	Cases/Total	1127/39957	1176/39927	1109/39944	1020/39937	970/39933	5402/199698	
	Model 0*	1.00(0.94-1.06)	1.17(1.11-1.24)	1.21(1.14-1.28)	1.20(1.13-1.28)	1.31(1.23-1.39)	1.08(1.06-1.11)	<0.0001
	Model 1†	1.00 (0.94-1.06)	1.13 (1.07-1.20)	1.16 (1.09-1.23)	1.15 (1.08-1.22)	1.25 (1.17-1.33)	1.07 (1.04-1.10)	<0.0001
	Additionally adjusted for SHBG‡	1.00 (0.94-1.06)	1.12 (1.06-1.19)	1.14 (1.08-1.21)	1.13 (1.06-1.20)	1.22 (1.14-1.30)	1.06 (1.04-1.09)	<0.0001
	Additionally adjusted for free testosterone‡	1.00 (0.94-1.06)	1.12 (1.06-1.19)	1.15 (1.08-1.22)	1.14 (1.07-1.21)	1.24 (1.16-1.32)	1.07 (1.04-1.09)	<0.0001
SHBG (per 10 nmol/L increase)	Cases/Total	681/36825	929/36804	1097/36805	1102/36786	1193/36786	5002/184006	
	Model 0*	1.00(0.93-1.08)	1.07(1.00-1.14)	1.09(1.03-1.16)	0.99(0.93-1.05)	0.96(0.91-1.02)	0.97(0.96-0.99)	<0.0001
	Model 1†	1.00 (0.93-1.08)	1.02 (0.96-1.09)	1.03 (0.97-1.09)	0.91 (0.86-0.97)	0.87 (0.82-0.92)	0.96 (0.94-0.98)	<0.0001
	Additionally adjusted for IGF-I‡	1.00 (0.92-1.08)	1.03 (0.96-1.10)	1.04 (0.98-1.10)	0.93 (0.88-0.99)	0.90 (0.85-0.96)	0.96 (0.95-0.98)	0.0001
Testosterone (per 5 nmol/L increase)	Cases/Total	1078/39820	1084/39817	1069/39788	1095/39816	1043/39798	5369/199039	
	Model 0*	1.00(0.94-1.06)	1.05(0.99-1.12)	1.05(0.99-1.12)	1.10(1.04-1.17)	1.11(1.05-1.18)	1.05(1.02-1.09)	0.005
	Model 1†	1.00 (0.94-1.06)	1.01 (0.95-1.07)	0.99 (0.94-1.05)	1.02 (0.96-1.08)	1.03 (0.96-1.09)	1.02 (0.98-1.06)	0.41
	Additionally adjusted for IGF-I‡	1.00 (0.94-1.06)	1.01 (0.95-1.07)	1.00 (0.94-1.06)	1.03 (0.97-1.09)	1.04 (0.98-1.11)	1.02 (0.98-1.06)	0.25
Free testosterone (per 50 pmol/L increase)	Cases/Total	1158/36601	1133/36600	1076/36600	873/36600	725/36600	4965/183001	
	Model 0*	1.00(0.94-1.06)	1.09(1.03-1.16)	1.18(1.11-1.25)	1.12(1.04-1.19)	1.24(1.15-1.33)	1.07(1.04-1.09)	<0.0001
	Model 1†	1.00 (0.94-1.06)	1.07 (1.01-1.13)	1.14 (1.07-1.21)	1.07 (1.00-1.15)	1.18 (1.10-1.27)	1.06 (1.03-1.08)	<0.0001
	Additionally adjusted for IGF-I‡	1.00 (0.94-1.06)	1.06 (1.00-1.13)	1.13 (1.06-1.20)	1.06 (0.99-1.13)	1.16 (1.08-1.25)	1.05 (1.03-1.08)	0.0001

* HRs are stratified by region (10 UK cancer registry regions) and age at recruitment (<45, 45–49, 50–54, 55–59, 60–64, and ≥65 years) and adjusted for age (underlying time variable).

†Model 0 additionally adjusted for Townsend deprivation score (fifths, unknown), racial/ethnic group (white, mixed background, Asian, black, other, unknown) , height (<170, ≥170–<175, ≥175–<180, ≥180 cm, unknown), lives with a wife or partner (no, yes), BMI (<25, ≥25–<30, ≥30–<35, ≥35 kg/m^2), cigarette smoking (never, former, light smoker, heavy smoker, current unknown, and smoking status unknown), alcohol consumption (non-drinkers, <1-<10, ≥10-<20, ≥20 g ethanol/day, unknown), and diabetes (no, yes, and unknown).

‡Model 1 additionally adjusted for hormone in categories of fifths and missing.

Abbreviations: BMI=body mass index; CI=confidence interval; HR=hazard ratio; IGF-I=insulin-like growth factor-I; SHBG=sex hormone binding globulin.

Supplementary Table S5: PheWAS associations of the IGF-I *cis*-SNP

Trait	Beta	P
Peak expiratory flow	-0.016	2.54E-11
Forced vital capacity	-0.012	1.48E-07
Impedance of arm right	0.010	2.28E-07
Impedance of arm left	0.010	1.07E-06
Impedance of whole body	0.010	1.08E-06
Forced expiratory volume in 1-second	-0.011	1.24E-06
Forced vital capacity, best measure	-0.012	1.31E-06
Forced expiratory volume in 1-second, best measure	-0.012	5.48E-06
Bring up phlegm or sputum/mucus on most days	-0.007	7.21E-06

*Source: PhenoScanner

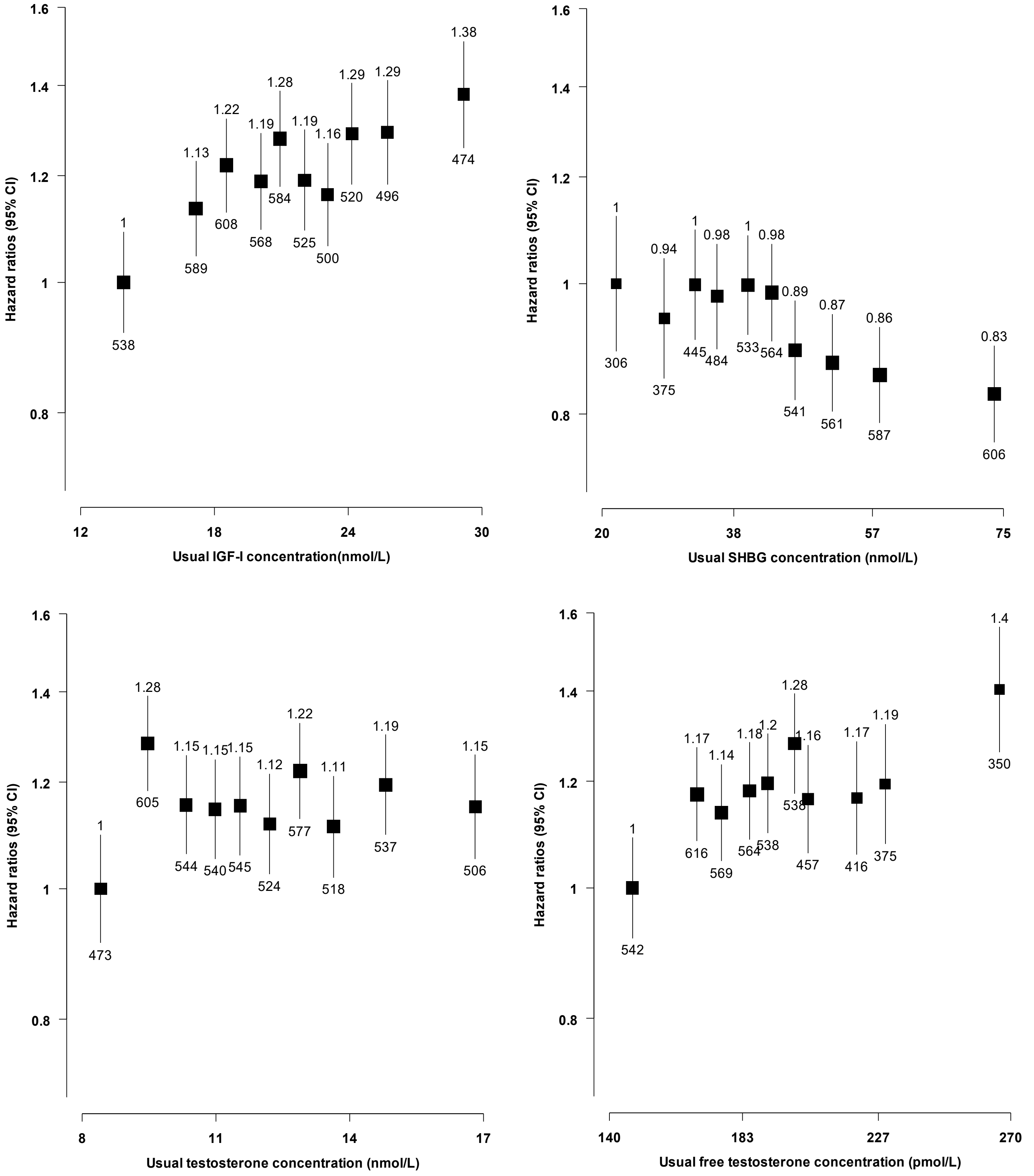

Supplementary Figure S1: Hazard ratio of incident prostate cancer by tenths of serum hormone concentrations in UK Biobank

HRs are stratified by region (10 UK cancer registry regions) and age at recruitment (<45, 45–49, 50–54, 55–59, 60–64, and ≥65 years) and adjusted for age (underlying time variable), Townsend deprivation score (fifths, unknown), racial/ethnic group (white, mixed background, Asian, black, other, unknown), height (<170, ≥170–<175, ≥175–<180, ≥180 cm, unknown), lives with a wife or partner (no, yes), BMI (<25, ≥25–<30, ≥30–<35, ≥35 kg/m^2), cigarette smoking (never, former, light smoker, heavy smoker, current unknown, and smoking status unknown), alcohol consumption (non-drinkers, <1-<10, ≥10-<20, ≥20 g ethanol/day, unknown), and diabetes (no, yes, and unknown). The boxes represent the HRs; the vertical lines represent the 95% CIs, with the size inversely proportional to the variance of the logarithm of the HR. The numbers above the vertical lines are point estimates for HRs, and the numbers below are the number of prostate cancer diagnoses.

Abbreviations: BMI= body mass index; CI= confidence intervals; HR= hazard ratio; IGF-I=insulin-like growth factor-I; SHBG=sex hormone binding globulin.

Factor and subset	PCa Cases/ Total	HR per 5 nmol/L increase (95% CI
**Age at diagnosis, years**		
≤ 65	2374/199039	0.98 (0.93, 1.04)
> 65	2991/199039	1.04 (0.99, 1.10)
		Heterogeneity: χ_1^2= 0.15 (p= 0.1457)
**Blood collection to diagnosis, years**		
≤ 4	2758/199039	1.01 (0.95, 1.06)
> 4	2611/199039	1.03 (0.97, 1.09)
		Heterogeneity: χ_1^2= 0.63 (p= 0.6255)
**Age at recruitment, years**		
≤ 60	1336/112777	0.98 (0.91, 1.06)
> 60	4033/86262	1.03 (0.98, 1.08)
		Heterogeneity: χ_1^2= 1.10 (p= 0.2948)
**BMI, kg/m²**		
<30	4177/147929	1.01 (0.97, 1.05)
≥ 30	1192/51110	1.05 (0.97, 1.15)
		Heterogeneity: χ_1^2= 0.82 (p= 0.3660)
**Smoking status**		
Former/never	4839/173088	1.01 (0.97, 1.06)
Current	498/24789	1.02 (0.90, 1.15)
		Heterogeneity: χ_1^2= 0.00 (p= 0.9454)
**Alcohol consumption, g ethanol/day**		
< 10	1794/69056	1.02 (0.96, 1.09)
≥ 10	3553/128960	1.01 (0.97, 1.06)
		Heterogeneity: χ_1^2= 0.03 (p= 0.8652)
**Educational status**		
No university degree	857/37209	1.05 (0.95, 1.15)
University degree	3333/125767	0.99 (0.94, 1.04)
		Heterogeneity: χ_1^2= 1.17 (p= 0.2804)
**Currently married/cohabiting**		
No	197/11649	1.03 (0.85, 1.24)
Yes	4259/152152	1.00 (0.96, 1.05)
		Heterogeneity: χ_1^2= 0.06 (p= 0.8040)
**Townsend index**		
< median (less deprived)	2991/99895	1.01 (0.96, 1.07)
≥ median (more deprived)	2372/98886	1.02 (0.96, 1.08)
		Heterogeneity: χ_1^2= 0.01 (p= 0.9252)
**Ethnicity**		
White	5117/187262	1.01 (0.97, 1.05)
Non-white	224/10709	1.12 (0.93, 1.35)
		Heterogeneity: χ_1^2= 1.11 (p= 0.2921)
**Height, cm**		
≤ 175	2831/98477	1.02 (0.97, 1.07)
> 175	2534/100397	1.01 (0.96, 1.07)
		Heterogeneity: χ_1^2= 0.02 (p= 0.8870)
**Diabetes**		
Yes	311/13499	1.23 (1.05, 1.44)
No	5039/184570	1.01 (0.97, 1.05)
		Heterogeneity: χ_1^2= 5.88 (p= 0.0153)
**Family history of prostate cancer**		
No	2097/94774	0.98 (0.92, 1.04)
Yes	695/14871	0.97 (0.87, 1.08)
		Heterogeneity: χ_1^2= 0.01 (p= 0.9252)
**Poor self-reported health**		
Yes	169/9488	1.08 (0.87, 1.35)
No	5177/188395	1.02 (0.98, 1.06)
		Heterogeneity: χ_1^2= 0.31 (p= 0.5755)
**IGF-I concentration**		
< median	2862/98989	1.00 (0.95, 1.05)
≥ median	2484/98979	1.05 (0.99, 1.11)
		Heterogeneity: χ_1^2= 1.40 (p= 0.2360)
**SHBG concentration**		
< median	2129/91532	1.11 (1.03, 1.20)
≥ median	3240/107507	1.04 (0.99, 1.09)
		Heterogeneity: χ_1^2= 1.92 (p= 0.1656)
**Overall ¹**	**5369/199039**	**1.02 (0.98, 1.06)**
**Overall ²**	**5369/199039**	**1.02 (0.97, 1.08)**

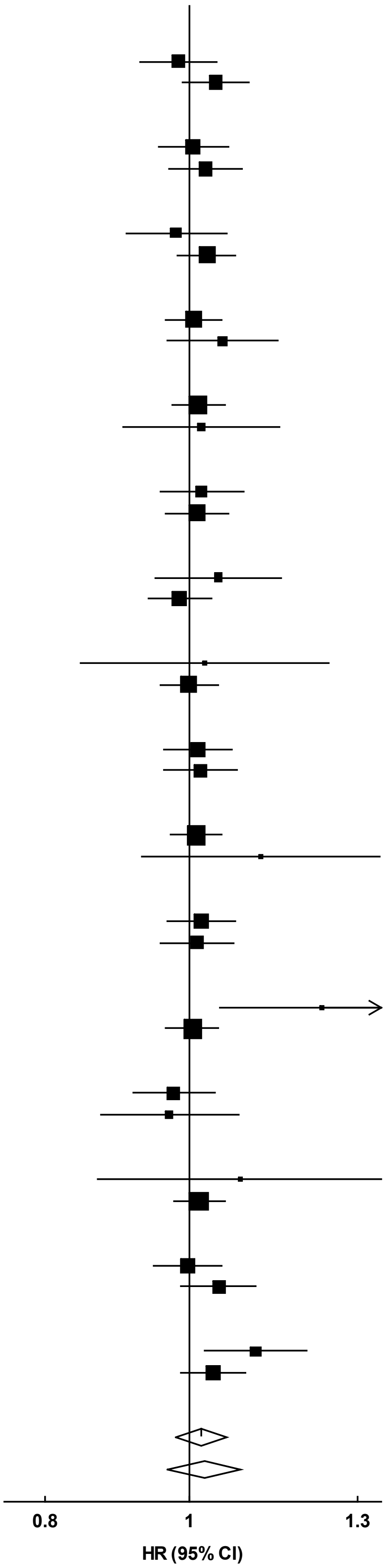

Supplementary Figure S2: Hazard ratio of incident prostate cancer per 5 nmol/L increase in serum total testosterone concentrations by subgroup in the UK Biobank

Cox models based on competing risks and compared the risk coefficients and standard errors in the two subgroups and tested using a χ^2 test of heterogeneity. For non-case specific factors, heterogeneity was assessed using a χ^2 interaction term.

HRs are stratified by region (10 UK cancer registry regions) and age at recruitment (<45, 45–49, 50–54, 55–59, 60–64, and ≥65 years) and adjusted for age (underlying time variable), Townsend deprivation score (fifths, unknown), racial/ethnic group (white, mixed background, Asian, black, other, unknown) , height (<170, ≥170–<175, ≥175–<180, ≥180 cm, unknown), lives with a wife or partner (no, yes), BMI (<25, ≥25–<30, ≥30–<35, ≥35 kg/m^2), cigarette smoking (never, former, light smoker, heavy smoker, current unknown, and smoking status unknown), alcohol consumption (non-drinkers, <1-<10, ≥10-<20, ≥20 g ethanol/day, unknown), and diabetes (no, yes, and unknown). The black boxes represent HRs, with the size inversely proportional to the variance of the logarithm of the HR, and the vertical lines represent 95% confidence intervals.

[1] Not adjusted for regression dilution bias.

[2] Adjusted for regression dilution bias.

Abbreviations: BMI= body mass index; CI= confidence intervals; HR= hazard ratio; PCa= prostate cancer; SD=standard deviation; SHBG=sex hormone binding globulin.

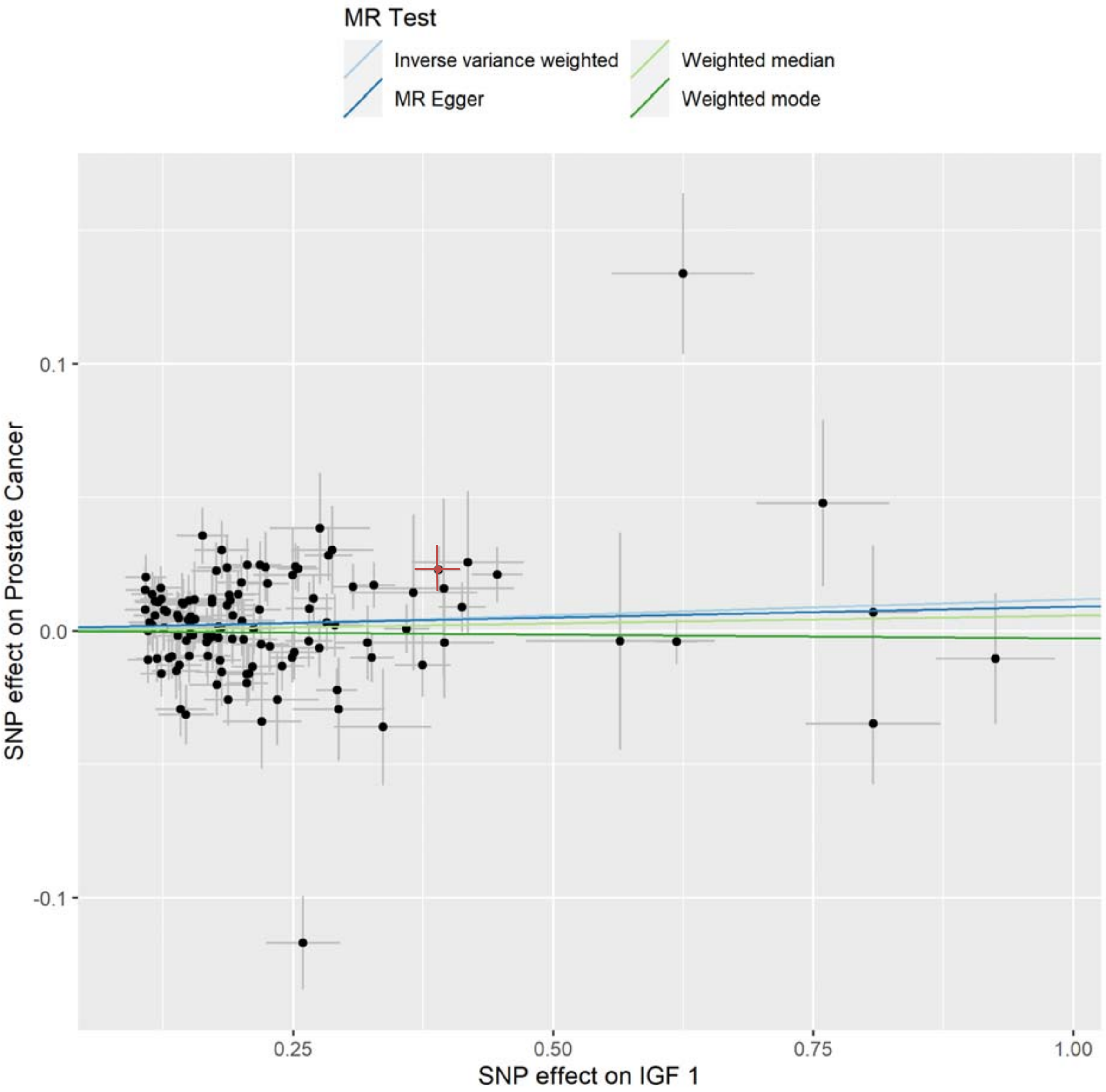

Supplementary Figure S3: Scatterplot of genetic associations with IGF-I against genetic associations with prostate cancer risk
Point estimates represent log odds ratios. Error bars represent 95% confidence intervals. *Cis*-SNP represented as the red datapoint. Abbreviations: IGF-I= insulin-like growth factor-I; SNP=single nucleotide repeat polymorphism.
